# Catapulting Tentacles in a Sticky Carnivorous Plant

**DOI:** 10.1371/journal.pone.0045735

**Published:** 2012-09-26

**Authors:** Simon Poppinga, Siegfried Richard Heinrich Hartmeyer, Robin Seidel, Tom Masselter, Irmgard Hartmeyer, Thomas Speck

**Affiliations:** 1 Plant Biomechanics Group, University of Freiburg, Freiburg im Breisgau, Germany; 2 Weil am Rhein, Germany; University of California, Berkeley, United States of America

## Abstract

Among trapping mechanisms in carnivorous plants, those termed ‘active’ have especially fascinated scientists since Charles Darwin’s early works because trap movements are involved. Fast snap-trapping and suction of prey are two of the most spectacular examples for how these plants actively catch animals, mainly arthropods, for a substantial nutrient supply. We show that *Drosera glanduligera*, a sundew from southern Australia, features a sophisticated catapult mechanism: Prey animals walking near the edge of the sundew trigger a touch-sensitive snap-tentacle, which swiftly catapults them onto adjacent sticky glue-tentacles; the insects are then slowly drawn within the concave trap leaf by sticky tentacles. This is the first detailed documentation and analysis of such catapult-flypaper traps in action and highlights a unique and surprisingly complex mechanical adaptation to carnivory.

## Introduction

Carnivorous plants catch and digest prey animals [1] and when their traps display movement they are termed ‘active’ [2]. Sundews (*Drosera* spp.) are well known to possess leaves with glandular emergences (tentacles) which secrete glistening, adhesive glue drops for attracting and capturing prey. Once an animal is caught and suffocated, digestive enzymes are produced by sessile glands and by the glue-tentacles, and the nutrients resulting from digestion become absorbed. Glue-tentacles and (in many species) the whole leaf blade can undergo slow bending movements to enfold and retain stuck prey which can take from several minutes up to several hours (active flypaper trap) [1]–[3]. *D. glanduligera*, a common and widespread annual from southern Australia [4], additionally features glue-free (non-sticky) snap-tentacles that can bend within a fraction of a second, similar to the speeds reported for Venus Flytrap snap-traps (*Dionaea muscipula*) [5]–[7]. This phenomenon was discovered by Richard (Tilbrooke) Davion in 1974, who published his field observations in 1995 and 1999 [8], [9] and mentioned that “… the dry pads are quite able to flick ants into the center of the traps.” Remarkably, these fascinating observations and interpretation received no consideration until 2010 [7], and trapping action in *D. glanduligera* has not been documented or investigated in depth until now. We show the first experimental evidence for the role of snap-tentacles in prey capture and provide a biophysical explanation for their fast motion.

## Materials and Methods

### Cultivation of Plants

Cultivation of *D. glanduligera* was accomplished in a temperate greenhouse of southwestern exposure. Approximately 300 seeds, harvested in April 2010, were sown in July 2010 but germinated with an extreme delay in October 2011 (approx. 200 seedlings, from which about 140 plants matured); further 40 seeds, harvested in 2009, were sown in July 2011 and germinated in November 2011 (12 seedlings, from which 7 plants matured). The soil used was a constantly wet peat/sand/pumice gravel mixture (2∶1:1). A 400 W metal-halide lamp (MT 400DL/BH, Iwasaki Electrics Co., Tokyo, Japan) was employed additionally for 9.5 hrs per day. Day-night temperature fluctuations ranged from 3°C–29°C at maximum in December 2011. Seedlings feature glue-tentacles from the first leaves and were fed with flaked fish food in 3–4 day intervals. From January 2012 on, larger plants with leaves of 2–3 mm in diameter were fed with fruit flies that were cut into halves, and plants with leaves of 3–4 mm in diameter were fed with complete flies.

### Prey Capture Experiments

We tested the ability of the snap-tentacles to fling prey using fruit flies (*Drosophila melanogaster*), which were purchased from Dehner garden center (Weil am Rhein, Germany) (flies with vestigial wings) (Video S1) or were provided by the Fischbach Laboratory of the University of Freiburg, Germany (wild-type flies) (Video S2 and S3). Flies were placed on the plant pots with featherweight forceps. Prey capture events were filmed with a HVR-Z5E HDV camcorder (Sony Co., Tokyo, Japan) (recording speed 25 fps) (Video S1) or with a Motion Scope Y4 high-speed camera (Redlake Inc., U.S.A.) (recording speed 2000 fps) in combination with a macro lens (Nikon AF Nikkor 28–105 mm) (Video S2). During high-speed camera recordings a techno light 270 cold-light source (Karl Storz GmbH & Co. KG, Tuttlingen, Germany) was used. Slower glue-tentacle movements were recorded with the cameras mentioned above (Videos S1 and S3).

**Figure 1:**
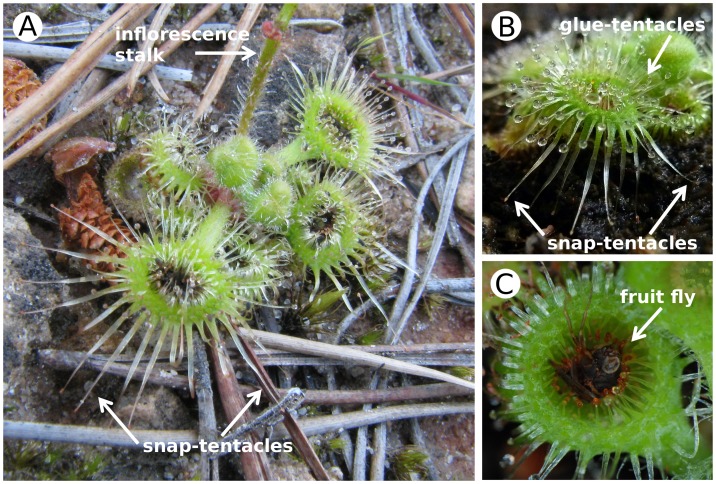
Trap leaves of *Drosera glanduligera*. (A) A naturally growing plant; note the peripheral, non-sticky snap-tentacles and the deeply concave trap leaves covered with glue-tentacles. (B) A cultivated plant; the snap-tentacles extend from the lamina margin. (C) A caught fruit fly; the prey is deeply drawn within the concave leaf blade.

### Snap-tentacle Motion Analyses and Image Evaluation

Six snap-tentacles were observed with a dissecting microscope Olympus SZX7, and their bending motions were recorded after manual triggering with a fine nylon thread on the tentacle heads using a high-speed camera and cold light source mentioned above (recording speed 2000 fps). We used a standard measuring tape (calibrated to 1 mm) to calculate distances. Video S4 was used for the calculations of velocity and acceleration. The detailed view of the bending of a snap-tentacle hinge-zone was recorded with the same high speed camera and cold light source in combination with an Axioplan light microscope (Carl Zeiss AG, Oberkochen, Germany) (Video S5).

For speed analyses of the tentacle head, the software Autopano (Version 2.5.1) was used for detecting corresponding feature points in subsequent images of Video S4. Afterwards, points on the head were selected manually and used for the calculation of its speed (R version 2.15.0) at each time step. The acceleration was obtained from the smoothening curve of the speed divided by the time of each interval. All images of the sequence were first averaged to obtain the background which was subsequently subtracted from each of the original images. Images corresponding to time shifts of 10 frames (5 ms) were chosen and added one after the other followed by normalizing of the obtained image after each step.

### Snap-tentacle Morphology

Snap-tentacles were excised at their bases with a razor blade and analyzed with a BX61 light microscope equipped with a DP71 digital camera and cell^∧^D 2.6 software (Olympus, Tokyo, Japan). 5 µm semi-thin transverse sections were produced with a custom-made rotating microtome after embedding the tentacles with Technovit7100 (standard procedure) (Heraeus Kulzer GmbH, Wehrheim, Germany). This involved 30 min deposition in isopropanol 50% and glycerine 99.5% (90∶10), followed by 30 min depositions in isopropanol of ascending concentration (70%, 90%). We applied toluidine-blue staining (infiltration for 2 min in toluidine C.I. 52040, 1 min washing with de-ionized water). Entellan (Merck KGaA, Darmstadt, Germany) was used for sealing the microscopy slides.

Scanning electron microscopy imaging was conducted with a SEM LEO 435 VP (Leica, Wiesbaden, Germany). Preparation of tentacles involved dehydration in methanol, critical point drying with a LPD 030 (Bal-Tec/Leica Mikrosysteme Vertrieb GmbH, Wetzlar, Germany), mounting on aluminium stubs with conductive adhesive tabs (Plano GmbH, Wetzlar, Germany), and gold coating (approx. 15 nm) with a Sputter Coater 108 auto (Cressington Scientific Instruments Ltd., Watford, England).

**Figure 2:**
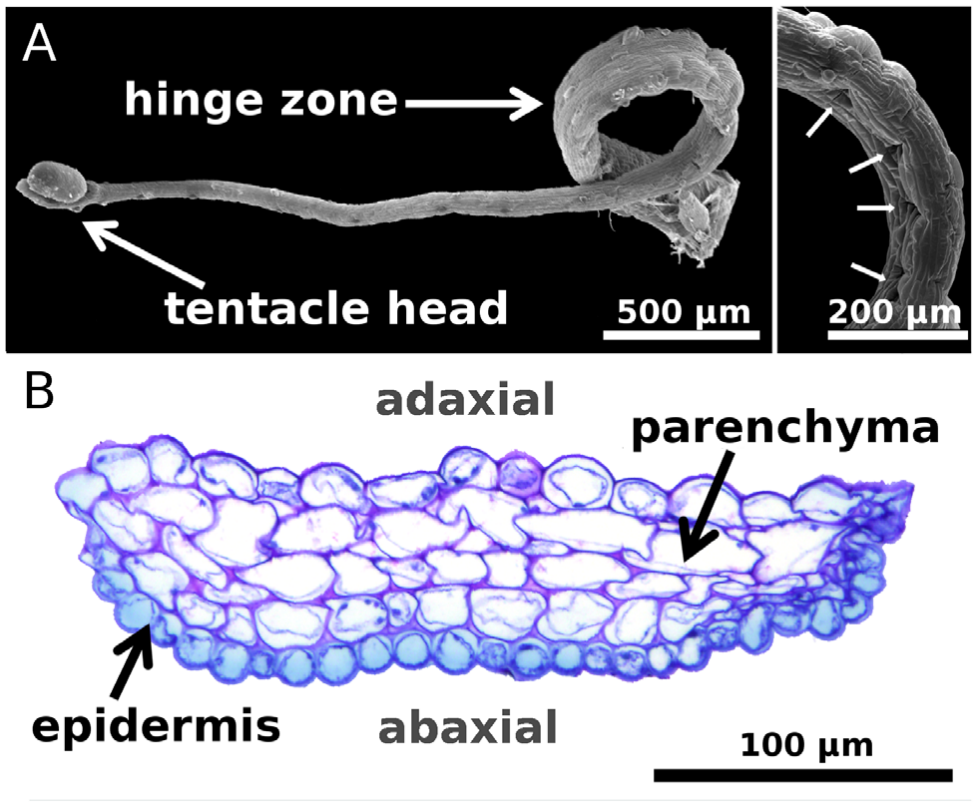
Snap-tentacle morphology and anatomy. (A) SEM micrographs of excised snap-tentacles. Left image: The bilateral symmetric tentacle is characterized by a gland raised above the terminal disc on the tentacle head. The gland does not produce mucilage. The hinge-zone is clearly visible. Right image: Fracture of adaxial epidermal cells in the hinge-zone (arrows) that presumably is due to local cell buckling caused by the compressive stresses acting on the adaxial side during the fast tentacle bending motion. (B) Transverse section of the hinge-zone, stained with toluidine. The abaxial epidermal cells are smaller than the adaxial epidermal cells. Both epidermis and parenchyma do not feature pronounced wall thickenings.

**Figure 3:**
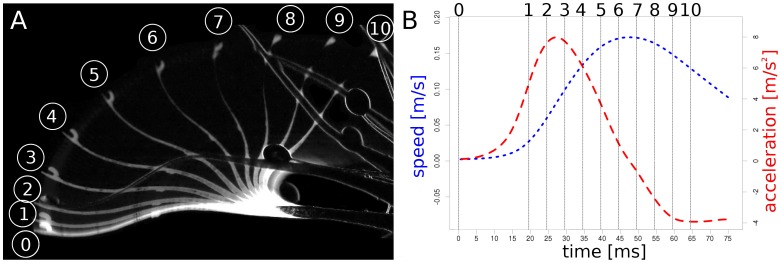
Snap-tentacle kinematics. (A) Each step between 1 and 10 depicts a 5 ms time interval. (B) Speed (blue) and acceleration (red) of the tentacle head during the bending motion (for a higher resolved curve see Fig. S1); the numbers correspond to the numbers depicted in (A).

## Results and Discussion

*D. glanduligera* grows as a rosette on the ground of up to 4 cm in diameter (Fig. 1a) and catches predominantly non-flying arthropods [10]. Each spoon-shaped trap leaf develops numerous glue-tentacles towards the centre and about 12–18 marginal snap-tentacles extending from the lamina margin (Fig. 1b). Both tentacle types are touch-sensitive, and their bending motions towards the centre of the trap are triggered by mechanical stimuli on the respective tentacle heads [3], [7]. Capture of walking prey takes place in two steps: First, animals that touch a snap-tentacle trigger its fast catapult-action and the prey is first lifted and then thrown onto the sticky central part of the leaf (Video S1 and S2). Subsequently, glue-tentacles draw the prey into the depression of the deeply concave leaf (Fig. 1c). This slower second step lasts about two minutes (Video S1 and S3). Further leaf blade movement (e.g., formation of a digestive groove) was not observed. The new observations confirm that the trap system employed by *D. glanduligera* is more complex than in other *Drosera* species relying solely on stickiness to catch prey and is thus more accurately termed a catapult-flypaper-trap. We observed that snap-tentacles are not triggered by vibrations of fruit flies already caught (Video S1–S3), hence are likely to become activated only by animals approaching the trap or escaping the glue (which was not observed, but is certainly possible).

Snap-tentacles are 6.3±2.2 mm (n = 11) long, bilaterally symmetric and each carry a raised gland (that does not produce mucilage) on the terminal disc [11] (Fig. 2). Triggering the head entails an initial post-stimulation phase without movement (about 400 ms). Snap-tentacles move by deformation of their hinge-zones [7] (Fig. 2a and b) which are situated next to the broadened tentacle base. Excised tentacles were observed to bend by 360 degrees, but when attached the motion is blocked halfway by the central part of the leaf. The duration of the smooth motion in some tentacles can be as fast as 75 ms (Video S4), with a maximum tentacle head velocity of 0.17 ms^−1^ and a maximum acceleration of 7.98 ms^−2^ (Fig. 3 and S1). However, other snap-tentacle movements were observed to last several seconds, suggesting that movement actuation is variable and possibly depends on the physiological condition or vigour of the plant or environmental factors such as temperature and humidity. Snap-tentacle motion is reported to be the fastest on very hot days [8].

We interpret the rapid change of tentacle curvature to be principally due to hydraulic forces within the tentacle and suggest two possible scenarios: 1) Rapid water transport occurs from cells of the adaxial half of the tentacle, which contract, to cells in the abaxial half, which thereby extend; or 2) actuation involves an active loss of turgor pressure in adaxial cells, followed by tentacle bending caused by prestress of the abaxial surface where epidermal cells are about half the size as those on the adaxial surface (Fig. 2b) and could store elastic energy. To elucidate the theoretical rate of hydraulic actuation we compared the duration of the fastest movement (τ = 75 ms) with the poroelastic time τp ∼ 16 ms according to Ref. [12], which characterizes the pressure equilibration time by fluid transport in the hinge-zone (smallest dimension ∼ 100 µm, Fig. 2b). τp is considerably lower than τ, which depicts that snap-tentacles do not necessarily require elastic instabilities to perform their fast motions. This is further corroborated by our observation that the transverse axis of the hinge-zone does not undergo a sudden geometrical change (curvature inversion) (Video S5) during the motion and that it consists of parenchymatous cells and epidermis without pronounced wall thickenings. Further verification requires investigation of the physiological aspects of motion and of the intrinsic mechanical properties of the hinge-zone tissue (e.g., the tendency of the epidermis to curve).

Snap-tentacle movement is not repeatable. This may be caused by fracture of epidermal cells of the hinge-zone (Fig. 2a) which is presumably a consequence of local cell buckling caused by compressive stresses acting on the adaxial side during the fast bending. As a short-lived annual with a growing season of about four months *D. glanduligera* grows fast and develops new leaves in intervals of three to four days, hence the catapulting tentacles can be regarded as ‘one shot devices’. *D. glanduligera* and sympatric, glue-trap only *D. erythrorhiza* both capture high numbers of springtails in their habitat [10], [13]. We interpret snap-tentacles (a) to increase the reach of a trap leaf and (b) to support capture of larger animals which might be strong enough to escape from the glue. Catapulting prey towards the trap centre, followed by further glue-tentacle movement, effectively brings prey into a more favorable position for retention, enzyme secretion, nutrient absorption, and protection from kleptoparasites [13]. Higher nutritional rewards resulting from more consistent capture and potentially larger prey could have acted as a selective advantage to favor evolution of snap-tentacles in *Drosera* [7] and snap-traps in the closely related Venus Flytrap (*Dionaea muscipula*) and Waterwheel Plant (*Aldrovanda vesiculosa*) [6].

From their sophisticated morphological/physiological adaptations (comprehensively reviewed in [14]) to the recent proof of carnivory in the genus *Philcoxia* [15]: Carnivorous plants are particularly interesting for plant biologists. Our analyses should encourage further research in different scientific disciplines. In physiology, especially electrophysiology, the touch-sensitive tissues, the way the tentacle's mechanical response is triggered, and the character of tentacle bending should be elucidated. This can be done e.g. by electrical irritation [16], [17], by investigation of the ion distribution within the respective tissues using ion-selective microelectrodes, by applying metabolic inhibitors, and by measurements of the change of turgor pressure. A recent and comprehensive approach on *Dionaea* snap-traps is given by Ref. [18]. In the domains of ecology, a detailed prey spectrum analysis could answer the question if the maximal prey mass is increased in this sundew compared to other species. Furthermore, experiments in the habitat should be undertaken that compare capture rates of plants whose snap tentacles have been clipped to plants with intact leaves. Such experiments will help to elucidate the actual advantage of having snap-tentacles. What’s more, the Droseraceae are also extremely interesting considering the different trapping mechanisms [3], [5], [19], [20], so that further analyses are very promising for shedding light on trap evolution [6].

## Supporting Information

Figure S1 **Detailed version of** **Figure 3b** **.** Speed (blue) and acceleration (red) of the tentacle head during the bending motion (Video S4).
(TIF)

Video S1 **Three capture events by snap-tentacles in real-time.** The prey animals are fruit flies with vestigial wings. The first recording also shows, in a time lapse sequence (x5), the subsequent glue tentacle movement and prey deposition towards the deeply concave center of the trap leaf. Recorded with 25 fps (MPG, 4.17 MB).
(MPG)

Video S2 **The capture of a wild-type fruit fly by a snap-tentacle.** Recorded with 2000 fps, played with 25 fps (MPG, 516 KB).
(MPG)

Video S3 **Time-lapse sequence (x25) of glue-tentacle movement after manual deposition of a wild-type fruit fly on the trap (MPG, 1.08 MB).**
(MPG)

Video S4 **The bending of a single snap-tentacle after manual stimulation with a nylon thread.** Recorded with 2000 fps, played with 25 fps (MPG, 1.23 MB).
(MPG)

Video S5 **A snap-tentacle hinge-zone during bending, after mechanical stimulation of the tentacle head with a nylon thread.** Recorded with 2000 fps, played with 25 fps (MPG, 926 KB).
(MPG)

## References

[1] Darwin C (1875) Insectivorous plants. London: John Murray.

[2] Lloyd FE (1942) The carnivorous plants. Waltham: Chronica Botanica.

[3] Williams SE (1976) Comparative sensory physiology of the Droseraceae - The evolution of a plant sensory system. Proc Am Philos Soc 120: 187–204.

[4] Lowrie A (1989) Carnivorous plants of Australia Vol. 2. Perth: University of Western Australia Press.

[5] Forterre Y, Skotheim JM, Dumais J, Mahadevan L (2005) How the Venus Flytrap snaps. Nature 433(27): 421–425. 15674293 10.1038/nature03185

[6] Gibson TC, Waller DM (2009) Evolving Darwin’s ‚most wonderful‘ plant: ecological steps to a snap-trap. New Phytol 183: 575–587. 19573135 10.1111/j.1469-8137.2009.02935.x

[7] Hartmeyer I, Hartmeyer SRH (2010) Snap-tentacles and runway lights. Carnivorous Plant Newsletter 39: 101–113.

[8] Davion R (1995) Now you see it - Now you don’t. Flytrap News 8: 17.

[9] Davion R (1999) That damned elusive Pimpernel. Flytrap News 13: 10.

[10] Verbeek NAM, Boasson R (1993) Relationship between types of prey captured and growth form in *Drosera* in southwestern Australia. Aust J Ecol 18: 203–207.

[11] Seine R, Barthlott W (1993) On the morphology of trichomes and tentacles of Droseraceae Salisb. Beitr Biol Pflanzen 67: 345–366.

[12] Skotheim JM, Mahadevan L (2005) Physical limits and design principles for plant and fungal movements. Science 308: 1308–1310. 15919993 10.1126/science.1107976

[13] Watson AP, Matthiessen JN, Springett BP (1982) Arthropod associates and macronutrient status of the red-ink sundew (*Drosera erythrorhiza* Lindl.). Aust J Ecol 7: 13–22.

[14] Król E, Plachno BJ, Adamec L, Stolarz M, Dziubinska H, et al (2012) Quite a few reasons for calling carnivores ‘the most wonderful plants in the world’. Ann Bot 109: 47–67. 21937485 10.1093/aob/mcr249 PMC3241575

[15] Pereira CG, Almenara DP, Winter CE, Fritsch PW, Lambers H, et al (2012) Underground leaves of *Philcoxia* trap and digest nematodes. PNAS 109(4): 1154–1158. 22232687 10.1073/pnas.1114199109 PMC3268334

[16] Williams SE, Pickard BG (1972) Receptor potentials and action potentials in *Drosera* tentacles. Planta 103: 193–221. 24481555 10.1007/BF00386844

[17] Williams SE, Pickard BG (1972) Properties of action potentials in *Drosera* tentacles. Planta 103: 222–240. 24481556 10.1007/BF00386845

[18] Escalante-Pérez M, Krol E, Stange A, Geiger D, Al-Rasheid KAS, et al (2011) A special pair of phytohormones controls excitability, slow closure, and external stomach formation in the Venus flytrap. PNAS 108(37): 15492–15497. 21896747 10.1073/pnas.1112535108 PMC3174645

[19] Ashida J (1934) Studies on the leaf movement of *Aldrovanda vesiculosa* L. I. Process and mechanism of the movement. Mem Coll Sci Kyoto Imp Univ Ser B9: 141–244.

[20] Poppinga S, Joyeux M (2011) Different mechanics of snap-trapping in the two closely related carnivorous plants *Dionaea muscipula* and *Aldrovanda vesiculosa* . Phys Rev E 84: 041928. 10.1103/PhysRevE.84.041928 22181196

