# Supplementary figures and images for: Catapulting Tentacles in a Sticky Carnivorous Plant
Source: PLoS One. 2012 Sep 26;7(9):e45735. doi: 10.1371/journal.pone.0045735 (PMC3458893; doi:10.1371/journal.pone.0045735)

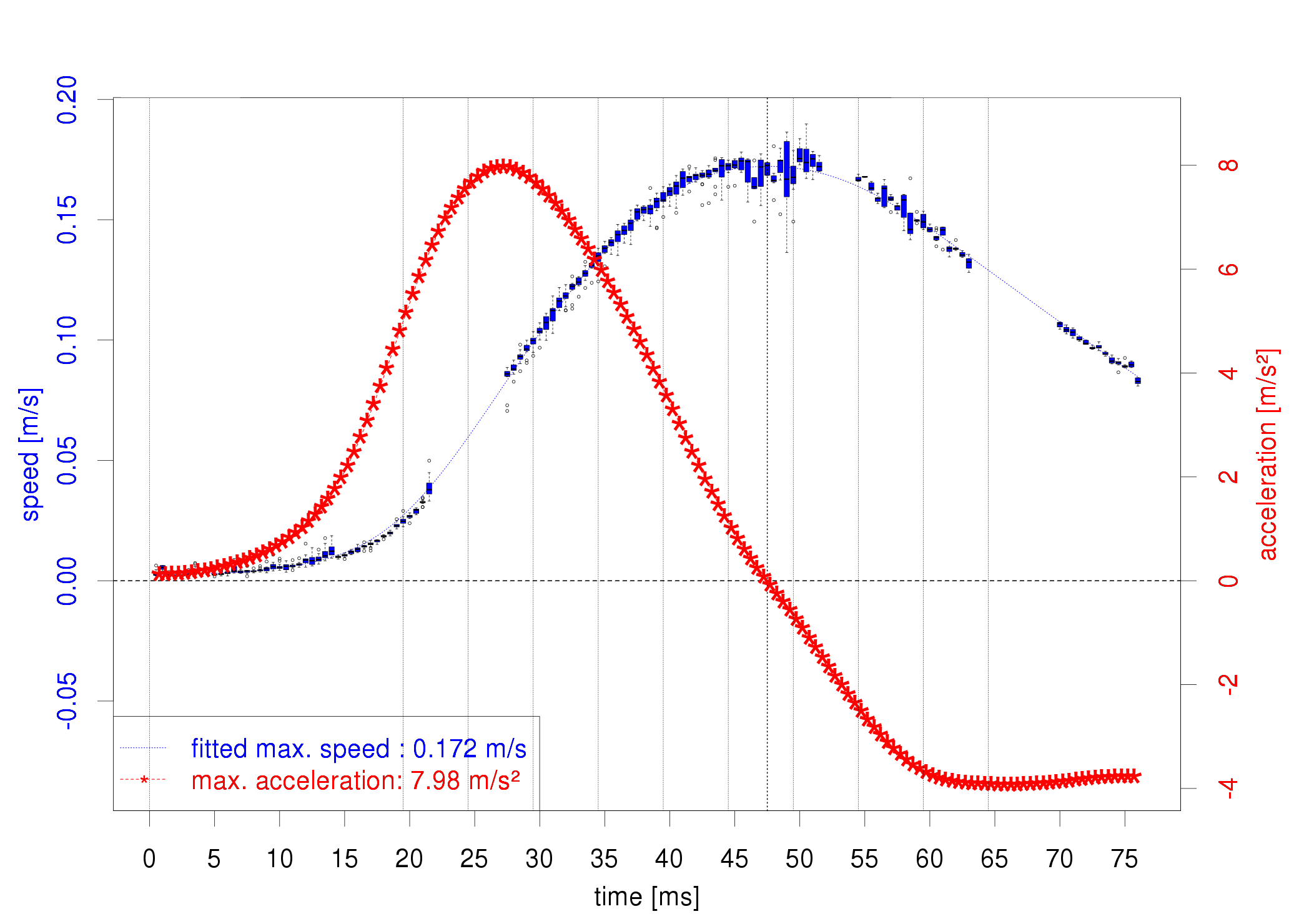

Supplement: Figure S1 — Detailed version of Figure 3b . Speed (blue) and acceleration (red) of the tentacle head during the bending motion (Video S4). (TIF) [file pone.0045735.s001.tif]
